# Questionnaire survey of physicians: Design and practical use in nephrology

**DOI:** 10.4103/0971-4065.53320

**Published:** 2009-04

**Authors:** Varun Agrawal, P. S. Garimella, S. J. Roshan, A. K. Ghosh

**Affiliations:** Department of Internal Medicine, William Beaumont Hospital, Royal Oak, Michigan, USA; 1 Division of Epidemiology and Community Health, School of Public Health, University of Minnesota, Minneapolis, USA; 2 HANSA MedCell, Mumbai, India; 3 Department of General Internal Medicine, Mayo Clinic, Rochester, Minnesota, USA

**Keywords:** Chronic kidney disease, graduate medical education, medical practice, questionnaire survey, residency

## Abstract

As medicine grows in complexity, it is imperative for physicians to update their knowledge base and practice to reflect current standards of care. Postgraduate training offers a golden opportunity for resident physicians to create a strong foundation of concepts in medicine. There is a need for assessing the knowledge of residents regarding established clinical practice guidelines and their perceptions regarding patient care and management. In this paper, we review how questionnaire surveys can be designed and applied to identify significant gaps in resident knowledge and inappropriate attitudes and beliefs. This evaluation has important implications for program directors who can then initiate measures to improve resident education. Such efforts during residency training have the potential of improving patient outcomes. We discuss the design of the questionnaire, its pre-testing and validity measures, online distribution, efficient response collection, data analysis, and possible future research. Finally, we illustrate this method of educational research with a questionnaire survey designed to measure the awareness of chronic kidney disease among internal medicine residents.

## Introduction

As medicine becomes more specialized, newer diseases and therapeutic modalities are discovered and their importance in clinical outcomes is recognized with active research. Clinical practice guidelines are designed by expert panels of scientific societies by a thorough review of the most up-to-date published literature. Though a few guideline recommendations are supported by clinical evidence, many others are expert opinions.[1] These guidelines, in general, are very helpful to guide the busy physician in managing common disorders and thus standardize the medical practice. The National Kidney Foundation developed a set of guidelines (K/DOQI Kidney Disease Outcomes Quality Initiative) for diagnosis and management of chronic kidney disease (CKD) that has been widely adopted by renal societies and physicians providing care to CKD patients.[2]

Postgraduate medical (residency) training offers the best opportunity to ensure accurate knowledge and clinical practice. Active involvement of the teaching faculty is vital to teach and inculcate appropriate practices. This helps generate a well-rounded graduate physician who is ready to practice medicine independently and is willing to learn new information. Throughout the course of the residency, the program needs to frequently assess the knowledge and attitude of residents towards important clinical conditions.[3] Such evaluations would not only highlight the strengths and weaknesses of individual residents, but would also provide program directors with the opportunities to identify gaps in resident knowledge and tailor the curriculum to address these issues. Similar educational assessments of practicing physicians can help national renal societies in disseminating accurate and up-to-date information through continued medical education thus allowing quality improvement.

**Figure 1:**
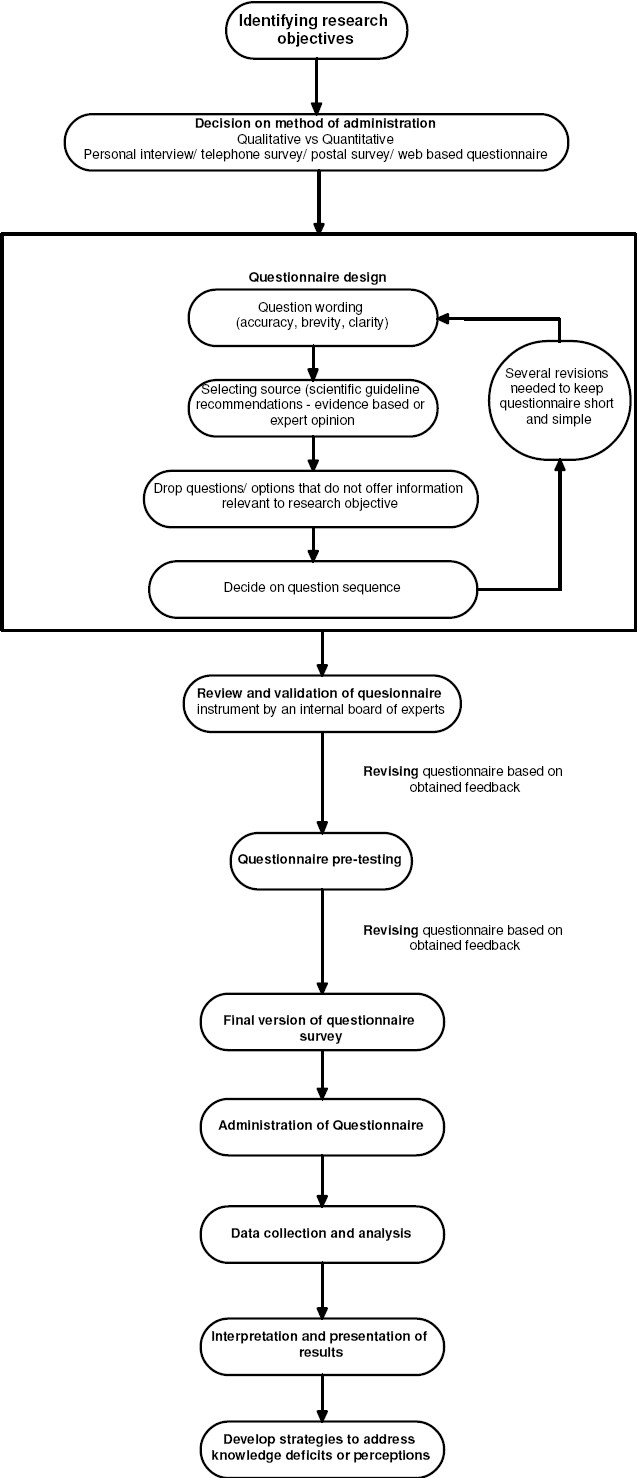
Steps in questionnaire survey design and administration

In this paper, we share our experience of questionnaire surveys in educational research in nephrology and discuss questionnaire design, pre-testing, validity establishment, and administration along with data collection and analysis [Figure 1]. We also provide an example illustrating the practical application of the above steps in an actual survey study testing medicine residents on knowledge of CKD.

## Identification of Study Question

The first step in a questionnaire survey is to identify the key research study question. A survey should be performed to identify knowledge deficits in diagnosis or management of important health issues, especially areas that the physician is to encounter commonly and has clinical implications. Questionnaire studies test for attitudes or perceptions of physicians that may help recognize inappropriate or wrongful behavior practice. The objective of the study and how the data would help improve physician healthcare practices with clinical implications should be clearly defined by the researcher. Diagnosis of CKD, monitoring of CKD progression and complications, and early nephrology referral are important aspects of CKD care provided by primary care physicians that are suboptimal and can benefit from major improvements.[4]

## Questionnaire Survey

Evaluation of a focus group can be performed through two paradigms - qualitative or quantitative. The qualitative survey employs open approach methods through active interviewing.[5] The researcher asks open-ended questions to the participants that are usually recorded on media. The recorded responses, being subjective, are analyzed in detail by trained reviewers to identify recurring themes and facilitate understanding of the beliefs of the participants or identify important barriers to improvement.[5] An example to illustrate this technique is provided in a qualitative survey of internal medicine residents to identify barriers towards implementation of evidence-based medicine.[6] The qualitative survey is a powerful exploratory research method, but is more labor-intensive and lacks the statistical rigor of quantitative methods.

Quantitative methods are more commonly used in medical surveys than qualitative methods to test almost any aspect of clinical medicine – knowledge of dyslipidemia management,[7] hypertension management,[8] tuberculosis management[9] and colorectal cancer screening.[10] The quantitative method typically employs multiple-choice questions and the responses can be scored thus allowing a quantitative assessment of the respondent's performance. It also has the added advantage of allowing a statistical analysis whereby the significance of associations can be tested and predictors of outcomes can be identified. Quantitative testing can also be done pre- and post-intervention to study its impact on physician knowledge, attitudes or behavior. Critical to the success of a questionnaire is its design, which we shall discuss in detail in the next few sections. Our intention is not to discuss questionnaire design for exam testing purposes, but solely for research purposes allowing improvement in knowledge or attitudes with interventions. Also, questionnaire survey methods can be applied to physicians at other levels of training and practicing physicians.

## Identification of Target Group

Before designing the survey, a target group needs to be carefully identified. This depends on the intent of the study, for example, if the study focuses on resident performance of cardiac auscultation skills,[11] then residents across different postgraduate years is a suitable study group. If the study is to compare the knowledge of transmission of *clostridium difficile* infection, then a study group comprising medicine residents, surgery residents, and intensive care unit nurses is appropriate. A study to evaluate stress or bullying in the health workplace is appropriately performed by surveying interns and postgraduate trainees.[12] A survey of private practitioners to evaluate practice patterns towards management of tuberculosis can provide very useful information to direct efforts at national collaboration for disease control.[13] If a study intends to compare how residents fare with hypertension management compared to their attendings, then a study group of residents and attendings would provide the suitable information. It is important to note that bias may occasionally result from the study group chosen, for example, surveying resident physicians at a national conference meeting may bias the findings as the responses are from physicians who are self-motivated and volunteered to attend the meeting and the responses cannot be generalized to all resident physicians. The researcher should identify the most appropriate target group to best serve the study purpose, and state the limitations clearly.

Once the focus group has been identified, the next important question is how best to sample the target group. Should all the medicine residents in a hospital be surveyed or should a random sampling be adequate? This is important to avoid wasting time and effort; if inadequate or excessive sampling is obtained that might also influence results. A helpful strategy is to calculate an appropriate sample size based on the results of pre-testing, as discussed later. The survey is then administered to this calculated number of respondents, as it is impractical to survey the entire population. A random sample of respondents should be chosen that best represent the population. Residents from university and community hospitals are to be included in the study sample to generalize the findings to the entire resident population.[14] For logistical reasons, sampling is usually done of residents in an institute or residency programs in the close vicinity of the study site to ensure as high a response rate as possible.[14] While attempting to identify a random sample, it is vital to be able to calculate the response rate (number of respondents/ number of persons in the study sample) as non-responder bias is a serious limitation of a survey that can confound the results dramatically.[15] In general, higher the response rate, greater is the confidence of generalizing the results to the population. A response rate of 50% is adequate, while a response rate greater than 70% is very good.[16]

## Designing the Questionnaire

Questionnaire design is the most critical part of the survey as the results and the interpretation of the findings depend on the questionnaire instrument.[17] Three key qualities of a questionnaire, accuracy, brevity and clarity are discussed below.

1. *Accuracy:* The questions should accurately reflect the concepts being tested and should be as direct as possible. There should be just one right answer to the question, unless it is a question with multiple right options. This ensures fairness and avoids confusion in the mind of the person who answers the question. The general theme of the questionnaire instrument should not be to trick the respondent, though some questionnaires benefit from negative responses to keep the examinee alert and reduce the possibility of a random guess. Clinical practice guidelines are useful sources for creating a questionnaire, as the testing points are evidence-based and recognized by an expert panel with recommendations graded based on the strength of current clinical evidence.
2. *Brevity:* While attempting to keep the survey as comprehensive as possible, it is equally important to keep the questionnaire short so that it can be completed within 15-20 min. Long and complex questionnaires attract fewer respondents and lead to a lower response rate.[15] The length of the questions and the options also should be short so as to be simple and easy to read and answer quickly.
3. *Clarity:* Strictly, there should be no ambiguity in the choices presented in the questionnaire. The questions and responses need to be very clear and any abbreviations used should be spelled out clearly at first use.

Multiple-choice questions often satisfy the above-mentioned qualities of the questionnaire. Including the option ‘I do not know’ is very helpful as it gives an accurate picture of the respondent's knowledge as they can now choose this option rather than make a guess. Opinions can be collected by the standardized Likert five-point scale (strongly disagree, disagree, neither agree nor disagree, agree, strongly agree). Questions should be centered on the research objective and be ethically appropriate. Besides questions on the subject content, a few questions need to be dedicated to data collection of the demographic characteristics of the respondents, especially postgraduate year distribution, age, gender distribution, prior clinical experience, and hospital setting. These data ensure that the study groups are well distributed and that the findings can be generalized to the physician population.

## Validation of Questionnaire

Once the questionnaire is designed, it needs to undergo an internal validation. Input from an expert panel of physicians is required to critically review the questionnaire and offer important feedback. Face validity is a subjective assessment where the independent reviewers of the questionnaire instrument ‘believe’ that the survey is adequate in assessing what it was intended for. Content validity is established when questions or options are agreed to have adequate performance by more than half of the reviewers.[18] Validation of the questionnaire is important to make fine adjustments to the questions and thus exclude ambivalence.

## Pre-Testing

A pilot study is very useful to study the responses, obtain feedback for further changes, and to ensure that the time to complete the survey is adequate. Usually, a small group of colleagues or residents in the program can be an appropriate sample to perform a pilot study. This can be done during a study lecture (noon conference) to ensure large number of responses and dedicated time to answer the questionnaire. From a statistical view, pre-testing allows the researcher to calculate the actual sample size needed for a complete study. The mean and standard deviation of the performance scores help the biostatistician calculate the sample size needed to detect significant intergroup differences. An example of pre-testing is provided in our case illustration.

## Questionnaire Administration and Collection of Responses

After incorporating further changes and feedback obtained from pre-testing, the questionnaire is finalized and ready to be sent out to the study sample [Table 1]. Traditionally, paper questionnaires are either handed out or mailed out to potential respondents. The questionnaires are printed, mailed out to the recipients with a self-addressed and stamped envelope. Besides the possibility of losing the mail in the postal service, the response rate is not adequate and can get expensive.[19] Repeat notifications need to be sent which again carry the above concerns. Paper questionnaires are labor-intensive, and the time and costs involved are often greater than those for an online survey.

**Table 1:** Comparison of survey methods in medical research

Survey method	Advantages	Disadvantages
Interviewer-administered questionnaires		
Face to face interviews	Useful for qualitative research Ambiguous responses can be immediately clarified	Labor- and cost-intensive Interviewers have to be trained Embarrassment might prevent accurate responses
Telephone interviews	Quicker to perform than face to face interviews Lesser manpower required Continuous response monitoring possible Follow-up easy to perform	Requires access to phone line Interviewers have to be trained Response rate may be low due to privacy concerns
Self-administered questionnaires		
In person	Immediate completion of the questionnaire by participants visiting study site	Only those in person are administered the questionnaire leading to selection bias
Postal questionnaire	Traditionally popular Can be answered at participants' convenience Avoids interviewer bias	Requires mailing addresses Long wait for and possible loss of responses Non-response rates can be high Bias due to self-selection
Web-based questionnaire	Fast and least expensive Lesser manpower required Anonymity ensured Repeated reminders possible Applicable to large scale studies	All participants need access to email and the internet Limited ability to generalize results as participants may be more educated Bias due to self-selection

In recent times, online questionnaires have become a faster method to perform a survey. Online questionnaires ensure quicker and accurate responses and reminders can be sent out easily.[20] Response rates can be as good as postal questionnaires, but there can be technical or methodological issues that may reduce its quality.[21] Online survey websites (www.surveymonkey.com or Google Docs) can help create online questionnaires that can be sent out en masse to a large number of respondents. The questions can be easily entered onto such survey programs and many question formats can be chosen (single answer, multiple answers, scale, check boxes or text entry). Numerous questions should not be crammed into one page as it gives the appearance of a busy questionnaire and may reduce response rate. After the questionnaire has been set up online, the survey program can send out the survey to different email addresses as desired. Furthermore, online programs can send out email reminders to try and obtain more responses. The responses obtained are neatly placed on a spreadsheet by the survey program that allows easy statistical analysis.

If the questionnaire is to be mailed out either on paper or electronically, a question arises as to how to reach out to the respondents. A good source is the site program director who usually has contacts with residency programs in the surrounding areas and can facilitate including more numbers of residents in the survey. Access to websites like the Association of Program Directors in Internal Medicine (APDIM) may offer resources including contact information of program directors of other residency programs.

A formal way of approaching the program director is to write a concise objective of the study including how the questions are to be answered and returned. If done online, the program director forwards the survey to his residents directly or through the chief residents. An incentive offered to the residents is very helpful to improve response rate. Educational CDs or pocket cards are useful and seem more ethical than financial incentives. The program director can be assured that a performance report of the program will be provided for internal review. Remember to be thankful to the physicians as they are volunteering their time and effort for your survey.

## Analysis and Reporting

Standard statistical analysis is performed with the help of a biostatistician to compare the performance scores. Each correct answer is usually given a score of 1, and incorrect or incomplete responses are scored zero. Regression analysis can be done to determine the predictors of final performance score. Reporting can be done using simple bar graphs to explain the distribution of the responses.

## Assessing Performance of the Questionnaire Instrument

We describe two commonly used indicators of the performance of the questionnaire. Item discrimination is a measure of how well the questions discriminate between the respondent with the highest and lowest score. For a question to be effective, participants with a high overall score should answer it correctly while those with a low score should not. If both those with a high and a low overall score answer a question similarly, the question has low item discrimination and should be considered for exclusion in future surveys. Item discrimination is calculated by subtracting the proportion of respondents who chose the right option in the highest tertile of final performance score from those in the lowest tertile. Questions with a higher discriminant index are more effective than those with a low index (>0.3 is considered adequate).

Cronbach's alpha is a test of reliability between the various sections of the questionnaire.[22] If multiple themes are being tested in the questionnaire (as discussed in the case illustration below), there needs to be some degree of correlation between the various sections so that they are all testing the participants on a particular topic. Statistical analysis with Cronbach's alpha gives a number that reflects correlations between different questionnaire sections (Cronbach's alpha value greater than 0.7 is adequate, while >0.9 is very good).

## Future Use of Questionnaire

The findings from the survey have important implications for directors of residency and nephrology programs as they can now identify the shortcomings of the training and institute educational interventions to overcome them. This may take the form of medical conferences and/or dedicated lectures by the nephrologists. Case-based learning may be more productive in improving knowledge of the residents. Similar avenues for improvement among practicing physicians (either general practitioners or nephrologists) may be identified by national societies like the Indian Society of Nephrology. Continued medical education content can be customized to address such knowledge deficits that may improve the overall delivery of care to CKD patients. Finally, quality improvement projects can take these findings and introduce measures to improve physician knowledge or change perceptions.

## Case Illustration

Chronic kidney disease (CKD) is a growing health problem with poor cardiovascular outcomes and progression to the need for dialysis. As CKD is being managed mainly by a primary care physician in the early stages, the National Kidney Foundation devised the Kidney Disease Outcomes Quality Initiative (KDOQI) guidelines for management of CKD that include definition and classification of CKD, evaluation of CKD, and management of hypertension, diabetes, hyperlipidemia, bone disease, nutrition, anemia, and cardiovascular disease in patients with CKD.[2] These guidelines recommend early detection of CKD, monitoring progression of CKD, assessment of complications, and timely referral to a nephrologist. A prior qualitative study showed poor awareness of these guidelines among primary care physicians.[23] We sought to assess how well physicians in training are aware of CKD and its management. We chose this target group because educational reforms in this early stage of the physician's career can effectively address knowledge deficits and practice patterns.

We reviewed the KDOQI guidelines and identified themes pertinent to an internist offering pre-end-stage renal disease care to the patient. The KDOQI guidelines offer a few recommendations that are evidence-based but most are the opinions of the expert panel. We carefully created a 16-item questionnaire that was reviewed by a panel of experts (seven nephrologists, one cardiologist and one internist) to establish face validity. The questions were multiple-choice and tested for knowledge of definition of CKD, classification of CKD, risk factors for CKD, laboratory evaluation of CKD, CKD management, management of complications of CKD, and referral to a nephrologist.

Next, we administered the questionnaire survey (paper form) to the residents in our program as part of the pilot study. The feedback obtained was critical in reducing some options, deletion of two questions and improving clarity of the questions. We then finalized the questionnaire and used a computer program to create the online survey. We obtained a grant to purchase access to the online program and also to buy educational pocket cards from the National Kidney Foundation.

We obtained the email addresses of all internal medicine program directors in the US from APDIM. We sent them a cover letter and the link to the survey. The program's participation was at the discretion of the program director. The survey program lacked the ability to tell how many program directors received the survey and how many accepted to participate. Once the residents got the links to the survey in their email, they answered it online quickly and effortlessly. After the study was completed, we sent them the educational pocket cards and answers to the survey.

We got 479 complete responses over a period of three weeks, thus reflecting the efficient nature of the online survey. Our study helped identify numerous gaps in the knowledge and perceptions among internal medicine residents towards CKD and its management.[24,25] In general, the feedback obtained was positive and very encouraging. We evaluated the performance of the questionnaire and found a few options that had a low discriminant index. These options will be deleted in the next edition of the questionnaire. Our questionnaire tested numerous aspects of CKD and Cronbach's alpha was calculated as 0.69. This is close to the recommended value (=0.70) and we consider this adequate for our questionnaire instrument as it tested numerous aspects of CKD identification and management. The inability to calculate the response rate was a serious limitation of the study but since this study was novel, it was recognized in national conferences and journals.

## Conclusion

Questionnaire surveys are an important assessment tool available to improve physician knowledge in nephrology and other fields of medicine. The various steps involved in the design, administration and analysis of a questionnaire are discussed in our paper and should be carefully implemented to ensure a high-quality questionnaire with good response rate and findings that can be generalized to the target population. Identification of knowledge deficits and misconceptions help the teaching physicians or national societies in directing efforts to change behavior and ultimately improve patient care and outcomes.
